# Vulnerabilities of children admitted to a pediatric inpatient care unit☆

**DOI:** 10.1016/j.rpped.2014.06.008

**Published:** 2014-12

**Authors:** Larissa Natacha de Oliveira, Márcia Koja Breigeiron, Sofia Hallmann, Maria Carolina Witkowski

**Affiliations:** a Hospital de Clínicas de Porto Alegre, Porto Alegre, RS, Brazil; b Universidade Federal do Rio Grande do Sul (UFRGS), Porto Alegre, RS, Brazil

**Keywords:** Health vulnerability, Children, Family, Inpatient Care Units

## Abstract

**OBJECTIVE:**

To identify the vulnerabilities of children admitted to a pediatric inpatient unit of a university hospital.

**METHODS:**

Cross-sectional, descriptive study from April to September 2013 with36 children aged 30 days to 12 years old, admitted to medical-surgical pediatric inpatient units of a university hospital and their caregivers. Data concerning sociocultural, socioeconomic and clinical context of children and their families were collected by interview with the child caregiver and from patients, records, and analyzed by descriptive statistics.

**RESULTS:**

Of the total sample, 97.1% (n=132) of children had at least one type of vulnerability, the majority related to the caregiver's level of education, followed by caregiver's financial situation, health history of the child, caregiver's family situation, use of alcohol, tobacco, and illicit drugs by the caregiver, family's living conditions, caregiver's schooling, and bonding between the caregiver and the child. Only 2.9% (n=4) of the children did not show any criteria to be classified in a category of vulnerability.

**CONCLUSIONS:**

Most children were classified has having a social vulnerability. It is imperative to create networks of support between the hospital and the primary healthcare service to promote healthcare practices directed to the needs of the child and family.

## Introduction

The legislation on the rights of children and adolescents,^1^ when not adhered to, leads the children and their families to a chain of events that affects not only their development, but also exposes them to vulnerabilities with consequent emergence of diseases. 

Vulnerabilities are the result of the interaction of a group of variables that determines a greater or lesser capacity to protect subjects from an injury, embarrassment, illness, or risk situation,^2^ and can be classified into individual, programmatic, and social levels. At the individual level, the knowledge about the diseases and the existence of behaviors that allow their occurrence is considered. At the programmatic level, the access to health services, their organization, the association between users and professionals of these services, as well as the prevention strategies and health controls are assessed. At the social level, the extent of the disease based on indicators that disclose the profile of the population in the affected area is assessed (access to information, expenses of social and health services, infant mortality rate, among others).^3^

Identification by and knowledge of the multidisciplinary team regarding such vulnerabilities that culminate in the health impairment of children and their families allows for providing greater completeness in health care, promoting the use of practices directed to these family's needs. Such consideration is proposed by the Extended Clinical Practice and Therapeutic Project (STP), in which a multidisciplinary team is committed to the patient, who is treated in a individualized way.^4^

Thus, the completeness of health actions in the context of STP "implies focusing on the political, social, and individual possibilities expressed by the individuals and by the collective, in their relations with the world, in their life contexts,^2^" and thus identifies and proposes targets for vulnerabilities found, in order to improve the quality of life of the children and their families. 

Therefore, the STP is a set of proposals that articulates therapeutic approaches for an individual or collective subject. This working model is a movement of co-production and co-management of the therapeutic process of these individual or collective subjects in situations of vulnerability, resulting from a discussion of the multidisciplinary team, with matrix support, if necessary, usually dedicated to more complex situations.^5^

The development of STP requires four distinct moments. The first step is the diagnosis, which should contain an organic, psychological, and social assessment, which allows a conclusion about the user's risks and vulnerabilities, also taking into account their perspective in relation to the health problem. The second step is the definition of goals in the short-, medium-, and long-term, which will be negotiated with the patient by the team member that has the best rapport with the patient. The third moment is the division of responsibilities, in which it is important to define the tasks of each member clearly. The fourth and final moment is the re-evaluation, at which time the evolution will be discussed and the necessary course corrections will be made.^5^

Considering the above, the objective of this study was to identify vulnerabilities of hospitalized children and of their families, which may be considered eligibility criteria for STP. Therefore, identifying the vulnerabilities of children and their families provides a better understanding and a greater benefit for the performance of the treatment plan and follow-up by the multidisciplinary team. 

## Method

This was a descriptive, cross-sectional, prospective cohort study, conducted in two medical-surgical units of a pediatric ward of Hospital de Clinicas de Porto Alegre, RS, Brazil. Clinical units cover different medical specialties and focus attention on the development of the methodology of care of hospitalized children and their families. 

The population consisted of children aged 30 days to 12 incomplete years. Inclusion criteria were any clinical diagnosis and a responsible adult in the family, older than 18 years. 

Sample size calculation was based on the rate of bed occupancy (80%) and monthly hospital admissions (80.5 hospital admissions) at the study units (data from the internal reporting service of Pediatric Inpatient Units provided by the Nursing Managers of these units). However, an error of 4% was considered, a confidence interval of 95%, and 20% loss, resulting in a sample of 136 children and their families.

Data collection occurred between April and September of 2013. The collection was performed using a tool developed by the researchers, which included closed questions related to demographic, economic, educational, and emotional aspects of the child and parent/guardian, as well as lifestyle habits, health status, and clinical care of the child. This tool was completed by the researchers who questioned the parent/guardian, at the child's bedside, during an interview with a maximum duration of 20 minutes. 

In addition to the interview with the child's parent/guardian, some data were collected from the patients' online medical records in order to obtain information on the diagnosis that led to the current hospitalization.

In order to assess the eligibility of vulnerabilities in the context of the child and his/her family, at the first moment, the dimensions that originated the items that contemplated the closed questions of the tool were indicated.

At a second moment, the presence or absence of vulnerabilities was defined, based on the interpretation of each item related to the dimensions, along with the concepts of vulnerability. Table 1 shows the dimensions of the sociocultural, socioeconomic, and clinical context, based on the questions of the data collection tool, in order to better discriminate the criteria regarding the presence of vulnerabilities in the sample. However, researchers defined the questions of the data collection tool as those that were closest to the concepts of vulnerability levels: individual, programmatic or social.^2^^,^ 3

**Table 1:**
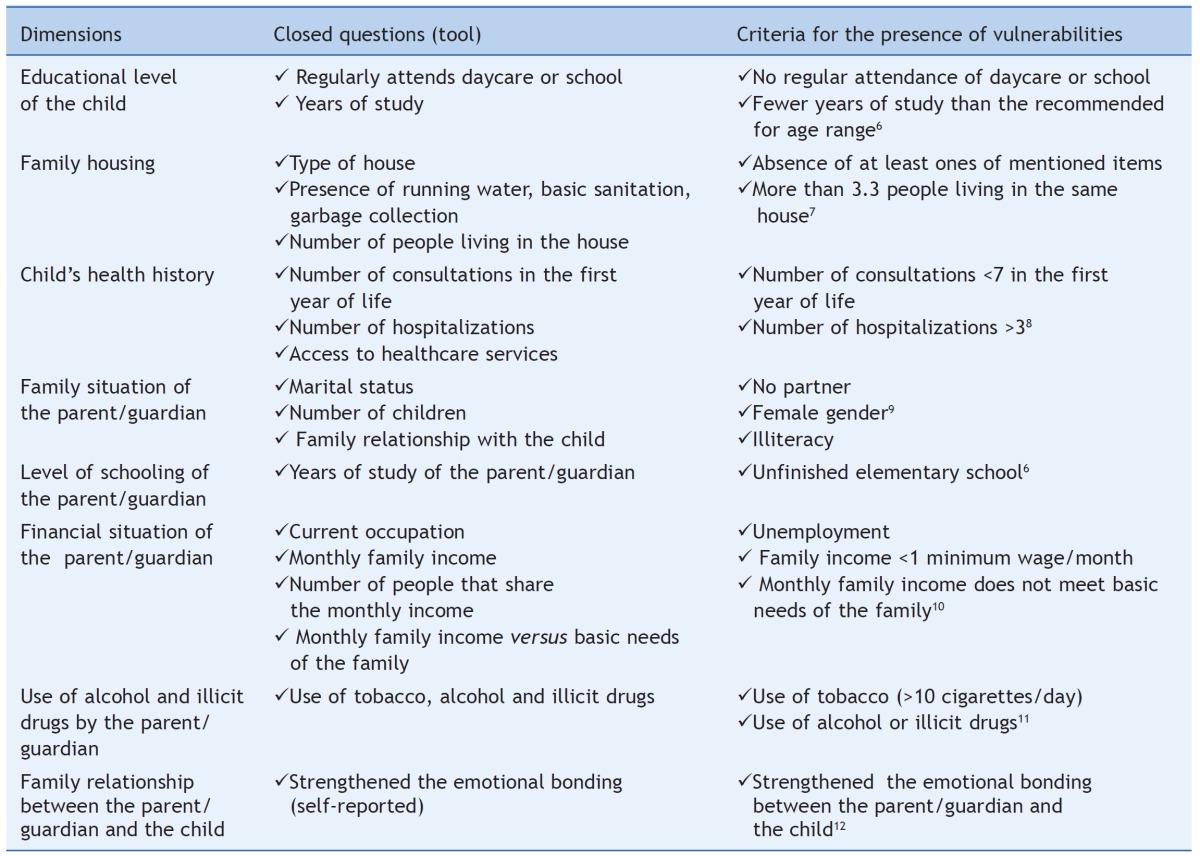
Dimensions and questions of the tool to identify the presence of vulnerabilities in the context of child and family. Porto Alegre, RS, Brazil, 2013.

Therefore, the dimensions "educational level of the child," "educational level of the parent/guardian," "living conditions of the family," "use of alcohol and illicit drugs by the parent/guardian," "emotional bonding between parent/guardian and the child," and "financial situation of the parent/guardian" would be inserted in individual vulnerability. In this regard, for the educational level, the fact of not regularly attending a nursery or daycare or having fewer years of schooling than what is recommended for the age group, considering the child, or illiteracy and incomplete elementary education, considering the parent/guardian, are factors that may expose the child/family to situations that lead to the health problem.^6^ From the same perspective, inadequate family housing or more than 3.3 individuals living in the same household^7^ also favor the occurrence of health problems. 

Moreover, the daily use of tobacco (>ten cigarettes/day) and alcohol or illicit drugs,^11^ as well as unemployment and family income that does not cover the basic needs of the family,^10^ can lead situations of domestic violence. Additionally, a weak emotional bonding between the parent/guardian and the child^12^ can lead to neglect in child care.

However, the dimension "health history of the child" was considered as programmatic vulnerability, in which the child's health problem can occur when health services are difficult to access, in terms of information and assistance.

As for the dimension "family situation of the parent/guardian", it was defined as social vulnerability. Single women, widows, and divorcees, who care for their children and assume the role of parent/guardian, are more likely to be exposed to social vulnerability.^9^

Nevertheless, regarding the eligibility criteria for STP, the researchers found that the context of the child/family would be eligible only if it were linked to four or more vulnerabilities, regardless of the type. Researchers justify the use of this criterion for the participation of children and their families in STP because it is infeasible to include all children in the project, considering its periodicity, as well as the number of professionals in the scenario of this study, and the fact that the vast majority of the children had at least some type of vulnerability.

Data were entered into a database of SPSS^(r)^ release 18.0 (Chicago, United States), with double entry of data for confirmation of records. Data were analyzed using descriptive statistics and shown as mean and standard deviation of the mean or median and interquartile range (25% and 75%), as well as absolute and relative frequencies. 

This study was approved by the Research Ethics Committee of Hospital de Clínicas de Porto Alegre, RS under protocol No. 130099. The parents/guardians were informed about the study objectives and signed the informed consent.

## Results

The overall characteristics of the sample are shown in Table 2. The sample consisted of 136 children admitted to the pediatric inpatient units, aged 33 months (range: 3.0 to 72.0), with a higher prevalence of males and white ethnicity. Most children did not attend daycare/school at the time of the interview and lived in brick houses with basic sanitation.

**Table 2:**
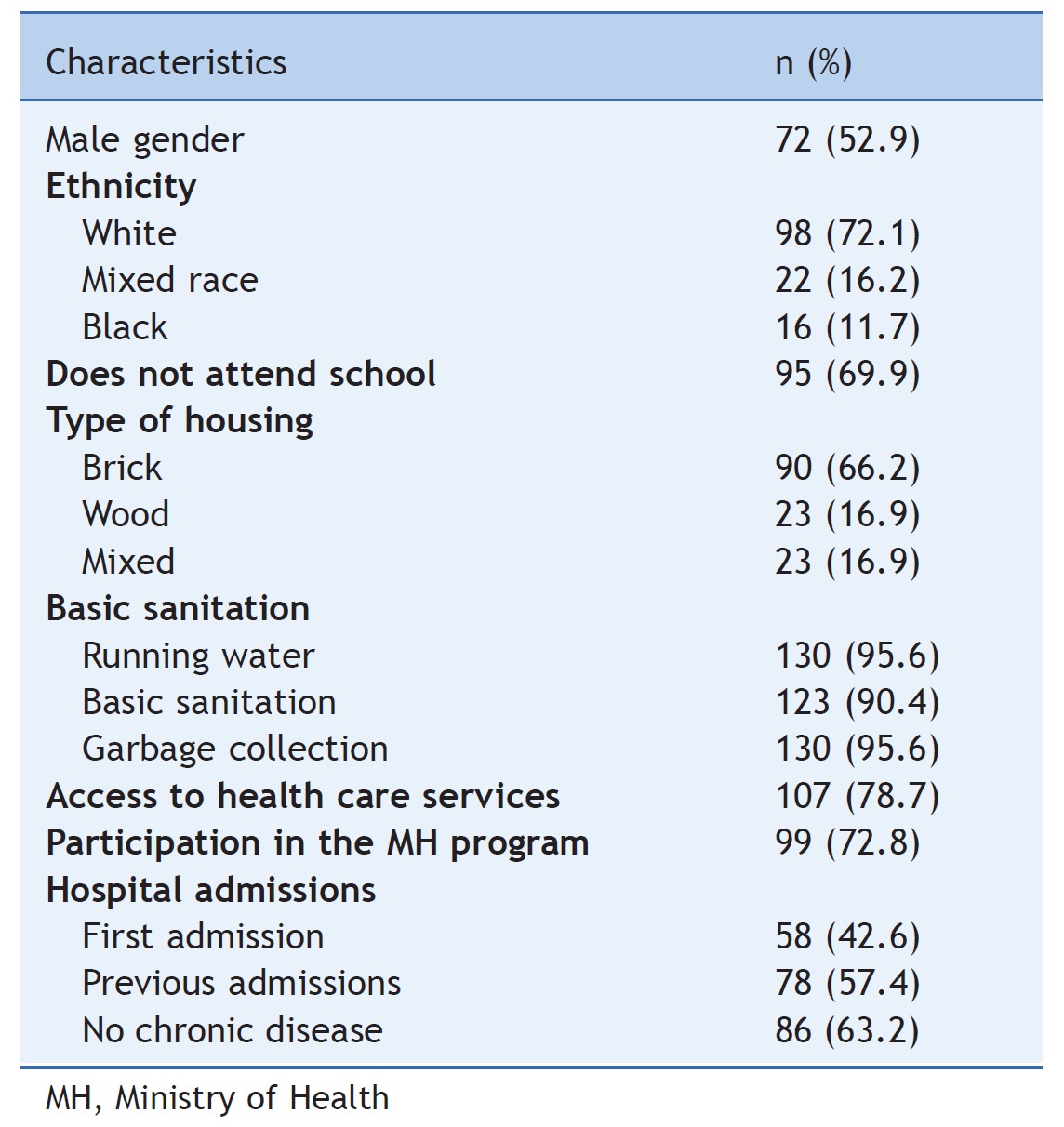
Sociodemographic characteristics of the 136 patients studied. Porto Alegre, RS, Brazil, 2013.

Regarding access to health services, the responses were positive in most cases, as well as the inclusion of children in the Ministry of Health programs at the Basic Health Units to which they belonged. However, most parents/guardians said that most of the children had prior hospitalizations, with 53.8% (n=42) of them with more than three previous hospital admissions. In most cases, the parents/guardians stated that the children did not have a clinical diagnosis of chronic disease. Considering the main clinical diagnosis of the current hospitalization, there was a prevalence of respiratory system diseases in 25.6% (n=35) of the sample. 

The characteristics of the children's parents/guardians are shown in Table 3. The age of the parent/guardian was 33 (range 25-38) years. Among these, 92.6% (n=126) were women, of which most were married or living with a partner and 61.8% (n=84) were housewives with no defined professional occupation. Of the 126 women, 103 reported being the biological mothers of the children and having other children, with a mean of 2.4 ± 1.4 children/woman. Regarding family income, most families had low income; however, most parents/guardians said that the income covered the basic needs of the family. Of the total sample, 39.7% (n=54) of the children lived with the father, mother and siblings, which represented an average of 3.8 ± 1.5 individuals per family who shared the family's monthly income. 

**Table 3:**
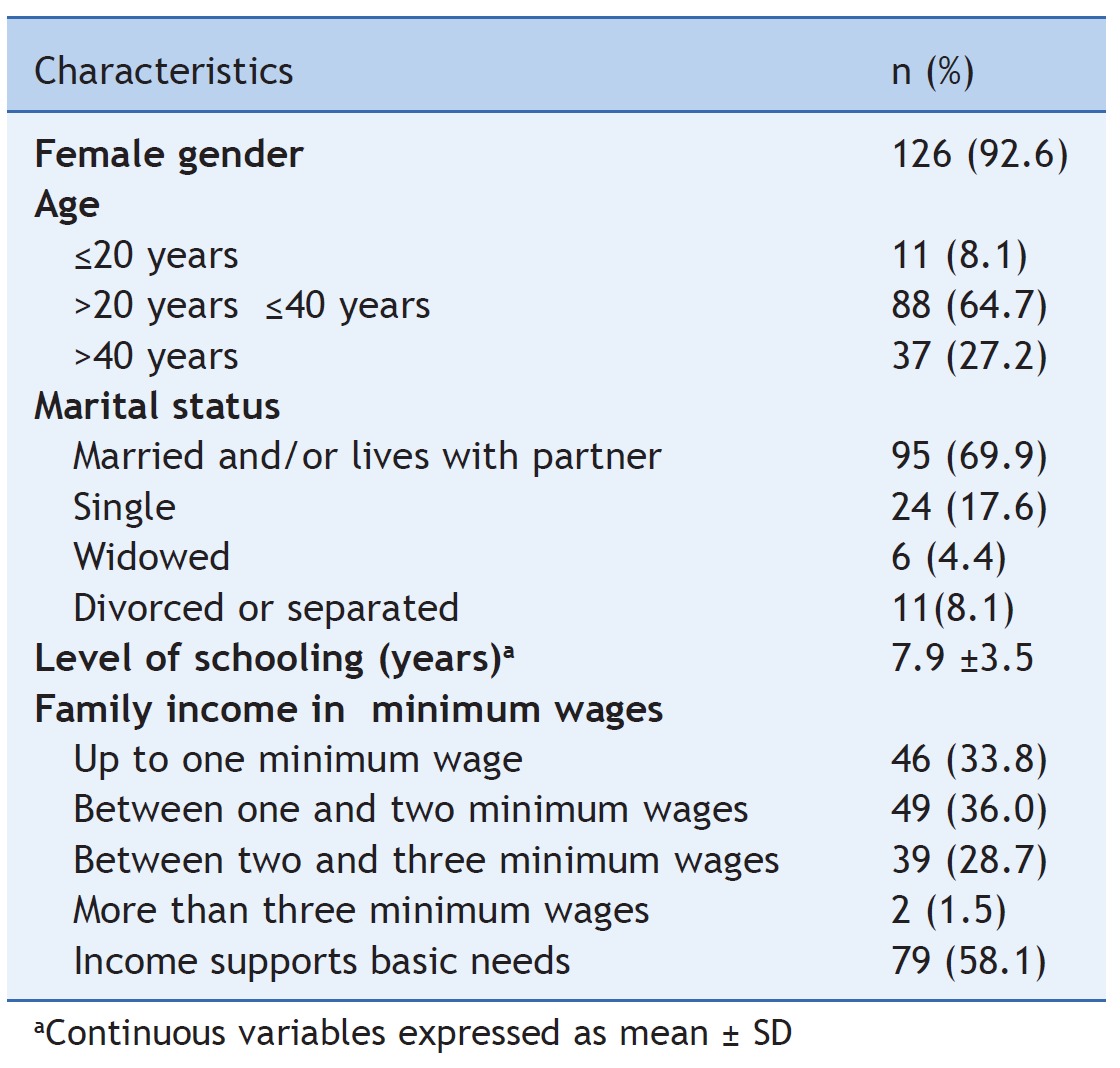
Sociodemographic characteristics of the parents/tutors of 136 patients. Porto Alegre, RS, 2013.

Concerning the assessed vulnerability (Table 4), 97.1% (n=132) of the families had at least one type of vulnerability. These were mostly related to the educational level of the parent/guardian, followed by the financial situation of the parent/guardian; health history of the child (prior presence of health disorder); family situation of the parent/guardian; use of alcohol, tobacco, and illicit drugs by the parent/guardian; family housing; education level of the child; and the emotional bonding with the child. Only 2.9% (n=4) of the children/families did not show any of the criteria for the presence of vulnerabilities, as shown in Table 1. As the eligibility criterion for STP was represented by the presence of at least four vulnerabilities, regardless of type, 23.5% (n=32) of children/families met this criterion.

**Table 4:**
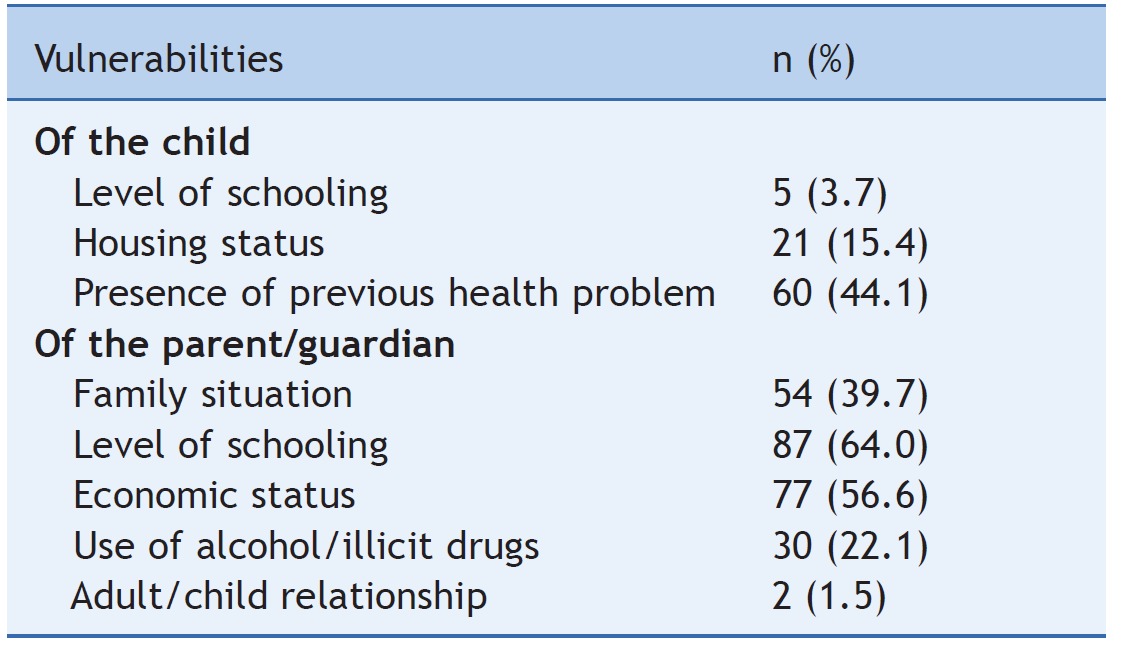
Vulnerabilities of the child/family (n=132). Porto Alegre, RS, Brazil, 2013.

## Discussion

This study was conducted with children admitted at hospital pediatric units and their families in relation to existing vulnerabilities regarding the child's health and disease process, both in the individual and the family context. However, considering that the aim of STP is to plan and provide comprehensive health care, the need to identify the vulnerabilities in the child's family context by the multidisciplinary team becomes imperative. Through STP, it is possible to create networks of support between the hospital environment and primary care, providing greater completeness in health care and promoting the use of practices directed to the needs of each child/family.

The vulnerabilities may result from different insertion or exclusion situations to which to children and their families are submitted, not restricted to a matter of social exclusion, but also as a matter of socialization and individualization.^13^

Among the existing vulnerability types, most children/families were classified as the social type. In this respect, there is a direct association between poverty and diseases, and between health and financial situation.^14^

Regarding the financial condition, families in vulnerable situations are exposed to inadequate conditions of education, food, housing, and quality of life. These factors result in the onset of diseases.^15^ Families whose monthly income did not cover the basic needs, as self-reported, presented the eligibility criteria for STP. These data corroborate the literature that indicates that the low purchasing power and the lack of financial resources of the families have a negative impact on child care and, thus, make them more susceptible to health problems.^14^

Therefore, the idea that the child's family income is a determinant in access to health services also predominates, and the situations of instability that permeate their daily lives appear as the cause of scarcity, ranging from those related to material goods to those that concern the autonomy of these individuals.^16^

The programmatic vulnerabilities are embedded into the context of child health regarding two main aspects: the development of health policies and the attitudes and collaboration of family members or parent/guardians, in order to make the home environment more adequate for health promotion.^14^ Changes in the daily routine organization are more evident in the daily life of a child admitted to a hospital, so that the family structure, school, or community must be included in this therapeutic process.

Considering that the family is mainly responsible for the child's development, it is essential to draw up a plan of care that has the family as its focus, considering the surrounding environment and the adequacy of the recommendations to their reality and limitations.^16^

As for the children who attended daycare or school, in the context of vulnerabilities, they were more often exposed to situations that met the eligibility criteria of STP at the individual level. In this regard, most of the sample was younger than 3 years, and at this age, staying in daycare and collective care institutions for young children is a growing tendency, both due to the need of the parents/guardians and due to work issues, as well as due to the importance of socialization and stimulation in child development. Thus, in such establishments, it becomes essential to provide staff training, parental guidance, and the involvement of health professionals to reduce both social and clinical problems for the children who, at some point in their lives, need to remain in these places.

On the other hand, the fact of regularly attending kindergarten and daycare can result in increased risk for diseases with consequent hospitalization, considering that these places have special epidemiological characteristics, for harboring a population with a characteristic profile and specific risk for transmission of infectious diseases. These epidemiological characteristics are related to the number of children that receive assistance collectively, favoring habits that facilitate the spread of diseases, as well as the presence of specific factors of the age range, such as the immaturity of the immune system.^17^

Individual vulnerability refers to the degree and quality of information that individuals have about health problems, their development, and practical application.^18^ This vulnerability is expressed by poor physical and psychological health status of the individual. Considering that a low educational level of the parents/guardians was predominant in the sample, the importance of the quality of information shared between the multidisciplinary team and the parent/guardian becomes evident. The low educational level of the parents/guardians of the children is directly related to the families' socioeconomic status, considering that a lower educational level of the parents/guardians is associated with lower employment opportunities, as well as worse living and health conditions.^14^^,^ 16

Regarding the questions related to basic sanitation, there was a predominance of children whose houses had no sewage system, running water, and garbage collection among those with STP eligibility, which is associated with children at higher nutritional risk.^19^ This demonstrates the importance of housing with adequate sanitation, considering that the lack of such conditions makes the environment unhealthy, and prone to contamination and disease proliferation.

Another important aspect that should be taken into consideration is the emotional bonding between the child and the parent/guardian. The emotional relationship between the family and the child is essential for the development of the foundations of the psychological formation for adulthood. Family situations where this relationship is fragile, especially when associated with other factors, may reflect a strong negative impact on child development, mainly up to school age.^12^

Although the use of a tool developed by the researchers and therefore not validated nationally should be acknowledged as a limitation of the study, , the results show that, at the individual vulnerability level, the eligibility criteria of STP were more evident, allowing the multidisciplinary team to design a monitoring plan geared to the specific needs of the families.

Most children/families showed some type of vulnerability, but not the minimum number required for eligibility and participation in STP. Only the fact that these children/families had some type of vulnerability would have justified a more individualized attention from the multidisciplinary team. Therefore, it is worth mentioning that the presence of only one vulnerability could be an indicative for the inclusion of these children/families into STP, differently from what was performed in the present study, in which the STP criterion was the presence of more than four vulnerabilities; this demonstrates the need for discussion regarding inclusion of new cases in STP in future studies. 

Thus, knowledge of the vulnerabilities present in the life of the child/family of the multidisciplinary team becomes of utmost importance, as it allows a more careful monitoring of cases and more comprehensive health care.
