# Supplementary material for: Vulnerabilities of children admitted to a pediatric inpatient care unit
Source: Rev Paul Pediatr. 2014 Dec;32(4):367–73. doi: 10.1016/j.rpped.2014.06.008 (PMC4311791; doi:10.1016/j.rpped.2014.06.008)
Supplement: Translation

# Translation: Vulnerabilidades de crianças admitidas em unidade de internação pediátrica☆

**Authors:** Larissa Natacha de Oliveira, Márcia Koja Breigeiron, Sofia Hallmann, Maria Carolina Witkowski

**Affiliations:** a Hospital de Clínicas de Porto Alegre, Porto Alegre, RS, Brasil; b Universidade Federal do Rio Grande do Sul (UFRGS), Porto Alegre, RS, Brasil

**Keywords:** Vulnerabilidade em saúde, Crianças, Família, Unidades de internação

## Abstract

**OBJETIVO:**

Identificar as vulnerabilidades de crianças admitidas em unidade de internação pediátrica de um hospital universitário.

**MÉTODOS:**

Estudo transversal, descritivo, realizado de abril a setembro de 2013. A amostra foi constituída por 136 crianças de 30 dias a 12 anos incompletos admitidas em unidades clínico-cirúrgicas de internação pediátrica de um hospital universitário, e seus responsáveis. Dados referentes ao contexto sociocultural, socioeconômico e clínico das crianças e suas famílias foram coletados por entrevista com o responsável da criança e por prontuário dos pacientes, sendo analisados por estatística descritiva.

**RESULTADOS:**

Do total da amostra, 97,1% (n=132) das crianças tinham pelo menos um tipo de vulnerabilidade, relacionadas, na sua maioria, ao nível de escolaridade do responsável da criança, seguida por: situação financeira do responsável, histórico de saúde da criança, situação familiar do responsável, uso de álcool, tabaco e drogas ilícitas pelo responsável, condições de moradia da família, nível de escolaridade da criança e vínculo do responsável com a criança. Apenas 2,9% (n=4) das crianças não apresentaram critérios que as classificassem como pertencentes a um tipo de vulnerabilidade, conforme pesquisado.

**CONCLUSÕES:**

A maioria das crianças foi classificada com vulnerabilidade social. A criação de redes de apoio entre o ambiente hospitalar e a atenção básica, promovendo a utilização de práticas direcionadas para as necessidades de cada criança e sua família, torna-se imperativa.

## Introdução

A legislação sobre os direitos da criança e do adolescente,^1^ quando não cumprida, leva a criança e sua família a uma cadeia de ocorrências que, não somente interferem no seu desenvolvimento, como também levam à exposição a vulnerabilidades com consequente aparecimento de agravos à saúde. 

Vulnerabilidades são o resultado da interação de um conjunto de variáveis que determina uma maior ou uma menor capacidade de proteção dos sujeitos a um agravo, constrangimento, adoecimento ou situação de risco,^2^ podendo ser classificadas em plano individual, programático e social. No plano individual, leva-se em consideração o conhecimento sobre o agravo e a existência de comportamentos que oportunizem a ocorrência deste. No plano programático, considera-se o acesso aos serviços de saúde, sua forma de organização, o vínculo entre os usuários e os profissionais destes serviços, assim como as ações de prevenção e controle de saúde. No plano social, examina-se a dimensão do adoecimento a partir de indicadores que revelem o perfil da população da área atingida (acesso à informação, gastos com serviços sociais e de saúde, coeficiente de mortalidade infantil, entre outros).^3^

A identificação e o conhecimento de tais vulnerabilidades, que culminam no agravo à saúde da criança e sua família, pela equipe multiprofissional, possibilita conferir maior integralidade às ações de saúde, promovendo a utilização de práticas direcionadas para as necessidades destas famílias. Tal consideração é proposta pela prática da Clínica Ampliada e do Projeto Terapêutico Singular (PTS), no qual a equipe multidisciplinar assume o compromisso com o sujeito doente visto de modo singular.^4^ Assim, a integralidade das ações de saúde no contexto do PTS "*implica em colocar no foco as possibilidades políticas, sociais e individuais expressas pelas pessoas e pelo coletivo, em suas relações com o mundo, nos seus contextos de vida* 2" e, assim, identificar e propor metas para as vulnerabilidades encontradas no intuito de melhorar a qualidade de vida das crianças e suas famílias. 

Deste modo, o PTS é um conjunto de propostas de condutas terapêuticas articuladas, para um sujeito individual ou coletivo. Este modelo de trabalho é um movimento de coprodução e de cogestão do processo terapêutico destes indivíduos ou coletivos em situação de vulnerabilidade, resultando de uma discussão da equipe multidisciplinar, com apoio matricial, se necessário, geralmente dedicado a situações mais complexas.^5^

O desenvolvimento do PTS requer quatro momentos. O primeiro momento é o diagnóstico, que deve conter uma avaliação orgânica, psicológica e social, que possibilite uma conclusão a respeito dos riscos e das vulnerabilidades do usuário, levando em consideração, também, sua visão diante do agravo à saúde. O segundo momento é a definição de metas em curto, médio e longo prazo, que serão negociadas com o sujeito doente pelo membro da equipe que tiver um vínculo melhor. O terceiro momento é a divisão de responsabilidades, em que é importante definir as tarefas de cada um com clareza. O quarto e último momento é a reavaliação, momento em que se discutirá a evolução e se farão as devidas correções de rumo.^5.^

Considerando o exposto, o objetivo deste estudo foi identificar vulnerabilidades de crianças internadas em unidade hospitalar e de suas famílias, que possam ser consideradas critérios de elegibilidade para o PTS. Deste modo, identificar as vulnerabilidades das crianças e de suas famílias propicia um melhor entendimento e um maior benefício para a condução do plano terapêutico e acompanhamento pela equipe multiprofissional. 

## Método

Estudo descritivo, de corte transversal, prospectivo, realizado em duas unidades clínico-cirúrgicas de internação pediátrica do Hospital de Clínicas de Porto Alegre/RS/Brasil. As unidades de internação abrangem diferentes especialidades e concentram atenção no desenvolvimento da metodologia do cuidado da criança internada e sua família. 

A população foi constituída por crianças de 30 dias a 12 anos incompletos. Como critérios de inclusão, foram considerados: quaisquer diagnósticos clínicos e responsável de reconhecimento familiar maior de 18 anos. 

O cálculo amostral foi baseado na média da taxa de ocupação dos leitos (80%) e de internações (80,5 internações) mensais das unidades em estudo (dados do relatório interno de atendimento das Unidades de Internação Pediátrica fornecido pelas Chefias de Enfermagem das referidas unidades). Entretanto, foi considerado um erro de 4%, intervalo de confiança de 95% e perda de 20%, resultando em uma amostra de 136 crianças e seus familiares. 

A coleta dos dados ocorreu entre os meses de abril e setembro de 2013. Para a coleta, foi utilizado um instrumento, elaborado pelos pesquisadores, que contemplou questões fechadas referentes aos aspectos demográfico, econômico, educacional e emocional da criança e do seu responsável, bem como hábitos de vida, situação de saúde e clínica da criança. Este instrumento foi preenchido pelos pesquisadores que interrogaram o responsável, junto ao leito da criança, em entrevista com duração máxima de 20 minutos. 

Além da entrevista com o responsável pela criança, alguns dados foram coletados do prontuário *online* dos pacientes, a fim de serem obtidas informações quanto ao diagnóstico que motivou a internação atual.

Para a elegibilidade das vulnerabilidades no contexto da criança e sua família, foram, em um primeiro momento, apontadas as dimensões que originaram os itens que contemplaram as questões fechadas do instrumento. Em um segundo momento, foi definida a presença ou não das vulnerabilidades, partindo da interpretação de cada item relacionado às dimensões, junto aos conceitos de vulnerabilidades. A tabela 1 mostra as dimensões no contexto sociocultural, socioeconômico e clínico, tomando por base as questões do instrumento de coleta de dados, com o intuito de melhor discriminar os critérios quanto à presença de vulnerabilidades na amostra em estudo. No entanto, os pesquisadores definiram as questões do instrumento de coleta dos dados como aquelas que mais se aproximavam aos conceitos dos planos de vulnerabilidades: individual, programática ou social.^2^^,^ 3 Deste modo, as dimensões "nível de escolaridade da criança", "nível de escolaridade do responsável", "condições de moradia da família", "uso de álcool e drogas pelo responsável", "vínculo do responsável com a criança" e "situação financeira do responsável" estariam inseridas em vulnerabilidade individual. Neste quesito, para o nível de escolaridade, não ter frequência regular à creche ou escola ou ter tempo de estudo menor do que é preconizado para a faixa etária, considerando a criança, ou analfabetismo e ensino fundamental incompleto, considerando o responsável, são fatores que podem expor a criança/família a situações que levam ao agravo à saúde.^6^ Nesta mesma perspectiva, condições inadequadas de moradia da família ou número maior que 3,3 pessoas residindo na mesma casa^7^ podem levar a comportamentos que, também, oportunizam a ocorrência de agravos à saúde. Ainda, o uso diário de tabaco (mais de 10 cigarros/dia) e de bebida alcoólica ou drogas ilícitas,^11^ bem como o desemprego e o fato da renda familiar mensal não comportar as necessidades básicas da família,^10^ podem levar a situações de violência doméstica. Também, o vínculo não fortalecido entre o responsável e a criança^12^ pode levar ao descaso no cuidado da criança. 

**Tabela 1:** Dimensões e questões do instrumento para identificar presença de vulnerabilidades no contexto da criança e sua família. Porto Alegre, RS, 2013

Por outro lado, a dimensão "histórico de saúde da criança" foi considerada como vulnerabilidade programática, na qual o agravo à saúde da criança pode acontecer quando os serviços de saúde são de difícil acesso em termos de informação e atendimento. 

Já a dimensão "situação familiar do responsável" foi definida como vulnerabilidade social. Mulheres solteiras, viúvas e divorciadas, que cuidam de seus filhos e assumem o papel de chefes de família, têm maior probabilidade de ficarem expostas à vulnerabilidade social.^9^

Entretanto, quanto aos critérios de elegibilidade para o PTS, os pesquisadores consideraram que o contexto da criança/família somente seria elegível se o mesmo fosse elencado a quatro ou mais vulnerabilidades, independentemente do tipo. Os pesquisadores justificam a utilização deste critério de participação da criança e sua família no PTS pelo fato de ser inviável contemplar todas as crianças no projeto, considerando a periodicidade em que este acontece, bem como, o número de profissionais na realidade deste estudo, sendo que a grande maioria das crianças apresentavam, pelo menos, alguma vulnerabilidade.

Os dados foram digitados no banco de dados do pacote estatístico *Statistical Package for Social Sciences* (SPSS) versão 18.0, com dupla digitação para confirmação dos resgistros. Os dados foram analisados pela estatística descritiva e apresentados por meio de média e desvio padrão da média ou mediana e intervalo interquartil (25% e 75%), e frequência absoluta e relativa. 

O presente estudo foi aprovado pelo Comitê de Ética e Pesquisa do Hospital de Clínicas de Porto Alegre/RS sob o nº de protocolo 130099. Os responsáveis pelas crianças foram esclarecidos sobre os objetivos da pesquisa e assinaram o Termo de Consentimento Livre e Esclarecido (TCLE). 

## Resultados

As características gerais da amostra estudada estão disponíveis na tabela 2. A amostra foi constituída por 136 crianças admitidas em unidades de internação pediátrica, com idade de 33 (variação: 3,0-72,0) meses, maior prevalência do sexo masculino e da raça branca. A maioria das crianças não frequentava creche/escola no momento da entrevista e residia em casas de alvenaria, com saneamento básico disponível. Quanto ao acesso a serviço de saúde, as respostas foram positivas na maioria dos casos, assim como a inserção das crianças em programas do Ministério da Saúde por cadastro nas Unidades Básicas de Saúde às quais pertenciam. Entretanto, os responsáveis afirmaram que a maioria das crianças apresentou internação hospitalar prévia, sendo que 53,8% (n=42) das mesmas com número maior do que três internações anteriores. Para a maioria dos casos, os responsáveis afirmaram que as crianças não possuíam diagnóstico clínico de doença crônica. Considerando o diagnóstico clínico principal da internação atual, houve prevalência para doenças relacionadas ao sistema respiratório em 25,6% (n=35) da amostra. 

**Tabela 2:** Características sóciodemográficas dos 136 pacientes analisados. Porto Alegre, RS, 2013

As características dos responsáveis pelas crianças são mostradas na tabela 3. A idade dos responsáveis das crianças foi de 33 (variação: 25-38) anos. Dentre estes, 92,6% (n=126) eram mulheres, a maioria em situação conjugal de casada ou residente com companheiro e 61,8% (n=84) eram do lar, sem atividade profissional definida. Das 126 mulheres, 103 afirmaram ser mães biológicas das crianças e possuír outros filhos, sendo a média de 2,4±1,4 filho/mulher. Quanto à renda familiar, a maioria das famílias encontrava-se em situação de baixa renda; entretanto, a maioria dos responsáveis afirmou que a renda comportava as necessidades básicas da família. Do total da amostra, 39,7% (n=54) das crianças conviviam com pai, mãe e irmãos, o que representou uma média de 3,8±1,5 pessoas por família que partilhavam a renda familiar mensal. 

**Tabela 3:** Características sociodemográficas dos responsáveis pelos 136 pacientes. Porto Alegre, RS, 2013

Quanto às vulnerabilidades pesquisadas (tabela 4), 97,1% (n=132) das famílias tinham pelo menos um tipo de vulnerabilidade. Estas vulnerabilidades estavam relacionadas, na sua maioria, ao nível de escolaridade do responsável, seguida por: situação financeira do responsável, histórico de saúde da criança (presença prévia de agravo à saúde), situação familiar do responsável, uso de álcool, tabaco e drogas ilícitas pelo responsável da criança, condições de moradia da família, nível de escolaridade da criança e vínculo do responsável com a criança. Apenas 2,9% (n=4) das crianças/famílias não apresentaram nenhum dos critérios para presença de vulnerabilidades, conforme indicado no Quadro 1. Como o critério de elegibilidade para o PTS, foi a presença de, no mínimo, quatro vulnerabilidades, independentemente do tipo, 23,5% (n=32) das crianças/famílias contemplaram este critério.

**Tabela 4:** Vulnerabilidades da criança/família (n=132). Porto Alegre, RS, 2013

## Discussão

Este estudo foi realizado com crianças internadas em unidades pediátricas e suas famílias em relação às vulnerabilidades existentes no processo de saúde e doença da criança, tanto no contexto individual quanto no contexto familiar. Entretanto, considerando ser objetivo do Projeto Terapêutico Singular (PTS) planejar e executar um cuidado integral à saúde, a necessidade de reconhecer as vulnerabilidades presentes no contexto familiar da criança, pela equipe multiprofissional, torna-se imperativa. Por meio do PTS, é possível a criação de redes de apoio entre o ambiente hospitalar e a atenção básica, conferindo maior integralidade nas ações de saúde e promovendo a utilização de práticas direcionadas para as necessidades de cada criança/família.

As vulnerabilidades podem ser consequência de diferentes modos de inserção ou de exclusão a que estão submetidas às crianças e suas famílias, não se restringindo apenas a uma questão de exclusão social, mas, também, como uma questão de socialização e individualização.^13^

Dentre os planos de vulnerabilidades existentes, a maioria das crianças/famílias foi classificada para o tipo social. Neste aspecto, há uma relação direta de pobreza com doenças e de saúde com situação financeira.^14^ No que diz respeito à condição financeira, as famílias em situação vulnerável são expostas às condições inadequadas de educação, alimentação, habitação e qualidade de vida. Tais fatores ocasionam o desenvolvimento de enfermidades.^15^ Apresentaram critérios de elegibilidade para o PTS aquelas famílias nas quais a renda mensal não comportou as necessidades básicas, segundo autorrelato. Tais dados corroboram a literatura que indica que o poder aquisitivo baixo e a falta de recursos financeiros das famílias geram um impacto negativo no cuidado à criança e com isso a tornam mais suscetível aos agravos à saúde.^14^ Deste modo, predomina também a ideia de que a renda dos pais ou cuidadores da criança é determinante no acesso aos serviços de saúde, e as situações de instabilidade que permeiam o seu cotidiano aparecem como causadores de carências, que vão desde as relacionadas a bens materiais até as que dizem respeito à autonomia destes sujeitos.^16^

As vulnerabilidades programáticas estão inseridas no contexto da saúde da criança, em dois aspectos principais: na elaboração de políticas de saúde e nas atitudes e na colaboração dos familiares ou responsáveis pelos cuidados, com a finalidade de tornar o ambiente familiar mais adequado para a promoção da saúde.^14^ As alterações na organização da rotina diária são as mais evidentes no cotidiano de uma criança internada em ambiente hospitalar, de modo que, a estrutura familiar, a escola ou a comunidade devem estar inseridas neste processo terapêutico. Considerando que a família é a principal responsável pelo desenvolvimento da criança, torna-se essencial a elaboração de um plano de cuidado que tenha a família como foco, preocupando-se com o ambiente que a cerca e com a adequação das orientações a sua realidade e as suas limitações.^16^

Quanto às crianças que frequentavam a escola, no contexto das vulnerabilidades, estas se encontraram mais expostas às situações que contemplavam os critérios de elegibilidade do PTS quanto ao plano individual. Nesta perspectiva, a maioria das crianças da amostra era menor de três anos, sendo, nesta faixa etária, a permanência em creches e instituições coletivas de cuidado a crianças pequenas uma tendência crescente, tanto por necessidade dos responsáveis e por questões de trabalho, quanto pela importância de socialização e estímulo no desenvolvimento das mesmas. Assim, em tais estabelecimentos, torna-se essencial o treinamento de funcionários, a orientação dos pais e o envolvimento de profissionais de saúde para a redução de problemas tanto de ordem social quanto clínica para as crianças que, em algum momento de suas vidas, necessitem permanecer nestes locais. Por outro lado, a frequência regular em creches e escolas pode proporcionar risco aumentado para doenças com consequente internação hospitalar, tendo em vista que tais estabelecimentos têm características epidemiológicas especiais, por abrigar população com perfil característico e com risco específico para a transmissão de doenças infecciosas. Estas características epidemiológicas estão relacionadas ao número de crianças aglomeradas recebendo assistência de forma coletiva, propiciando hábitos que facilitam a disseminação de doenças, além de também apresentarem fatores específicos da idade, tal como a imaturidade do sistema imunológico.^17^

A vulnerabilidade individual se refere ao grau e à qualidade da informação que os indivíduos dispõem sobre os problemas de saúde, sua elaboração e aplicação na prática.^18^ Esta vulnerabilidade é expressa por condições deficitárias de saúde física e psicológica do próprio indivíduo. Considerando que a baixa escolaridade dos responsáveis foi predominante na amostra, fica evidente a importância da qualidade de informações compartilhadas entre a equipe multiprofissional e o responsável. A baixa escolaridade dos responsáveis pelas crianças está diretamente relacionada à situação socioeconômica das famílias, considerando que o menor grau de instrução dos responsáveis se associa a menores oportunidades de emprego, assim como também a piores condições de vida e saúde.^14^^,^ 16

Nas questões relacionadas ao saneamento básico, houve predomínio de moradias das crianças que não apresentavam rede de esgoto, água encanada e coleta de lixo, para aquelas com elegibilidade para o PTS e se associa às crianças com maior risco nutricional.^19^ Isto demonstra a importância das moradias possuírem adequado saneamento básico, tendo em vista que a carência de tal condição torna o ambiente insalubre e propício a contaminações e proliferação de doenças.

Outro aspecto importante que deve ser levado em consideração é o vínculo entre a criança, o responsável e sua família. A relação afetiva familiar com a criança é imprescindível para o desenvolvimento de bases de formação psicológica para a fase adulta. Situações familiares em que há vínculo frágil, principalmente associado a outros fatores, podem refletir em forte impacto negativo no desenvolvimento da criança, principalmente até a idade escolar.^12^

Embora deva se reconhecer, como limitação do estudo, a utilização de um instrumento elaborado pelos pesquisadores, e, portanto, não validado nacionalmente, os resultados mostram que, no plano de vulnerabilidade individual, os critérios de elegibilidade do PTS foram mais evidenciados, propiciando que a equipe multiprofissional pudesse desenhar um acompanhamento voltado às necessidades e singularidades das famílias. A maioria das crianças/famílias apresentou alguma vulnerabilidade, mas não apresentou o número mínimo destas para elegibilidade e participação no PTS. Somente pelo fato destas crianças/famílias apresentarem alguma vulnerabilidade já seria justificável uma atenção mais individualizada da equipe multiprofissional. Desta maneira, torna-se importante ressaltar que a presença de apenas uma vulnerabilidade poderia ser indicativa para a inserção no PTS destas crianças/famílias, diferente do que foi realizado no presente estudo, na qual a presença de mais de quatro vulnerabilidades foi critério para o PTS, mostrando a necessidade de discussão quanto aos critérios relacionados à inserção no PTS de novos casos em estudos futuros. Assim, o conhecimento das vulnerabilidades presentes na vida da criança/família pela equipe multiprofissional torna-se de extrema importância, pois possibilita um acompanhamento mais criterioso dos casos e a realização de assistência à saúde de forma integral.
